# Gallic acid and metformin co-administration reduce oxidative stress, apoptosis and inflammation via Fas/caspase-3 and NF-κB signaling pathways in thioacetamide-induced acute hepatic encephalopathy in rats

**DOI:** 10.1186/s12906-023-04067-9

**Published:** 2023-07-25

**Authors:** Ehsan Khedre Mohamed, Dawlat Mohamed Hafez

**Affiliations:** 1 Biochemistry department, Egyptian DRUG AUTHORITY (EDA), formerly National Organization of Drug Control and Research (NODCAR), Giza, Egypt; 2 Histology department, Egyptian DRUG AUTHORITY (EDA), formerly National Organization of Drug Control and Research (NODCAR), Giza, Egypt

**Keywords:** Gallic acid, Metformin, Brain injury, NF-κB, FAS, Caspase-3

## Abstract

**Background:**

Hepatic encephalopathy (HE) is a consequence of chronic or acute liver diseases. This study evaluates the combined effect of gallic acid (GA), and metformin (Met) on the liver and brain damage associated with HE.

**Methods:**

Acute HE was induced by a single dose of thioacetamide (TAA) (300 mg/kg) as an I.P. injection. Treated groups received GA group (100 mg/kg/day, *p.o*), Met (200 mg/kg/day, *p.o*), or their combination for 25 consecutive days before TAA injection.

**Results:**

The administration of TAA induced various biochemical and histopathological alterations. In contrast, treatment with GA either alone or combined with Met resulted in improved liver functions by the significant reduction in serum ALT, AST, and ALP activities, and ammonia levels. Inflammatory mediators; TNF-α, IL-6, and NFkβ levels were decreased by these treatments as well as apoptotic cascade via down-regulation of FAS and caspase-3 (CASP-3) expression in hepatic tissues. Furthermore, GA and Met either alone or combined protected the liver and brain tissues from damage by increased glutathione concentration while decreasing malondialdehyde. In addition, it was accompanied by the improvement of the brain neurotransmitter profile via the restoration of norepinephrine, dopamine, and serotonin levels. Based on our data, this is the first study to report a novel combined hepatoprotective and cognitive enhancing effect of GA and Met against TAA-induced acute liver and brain injury.

**Conclusion:**

GA and Met combination resulted in a prominent improvement in HE complications, relative to monotherapy. Both agents potentiated the antioxidant, anti-inflammatory, and anti-apoptotic effects of each other.

## Introduction

Thioacetamide (TAA) is a synthetic chemical compound that has many applications as a chemical stabilizer in laboratories, as an organic solvent in paper, textile, and leather industries, and as a fungicidal agent [1]. It is considered a potent carcinogen and is utilized successfully as a hepatic encephalopathy (HE) model in rats via administration of a single dose of 300 mg/kg, I.P. Cytochrome P450 2E1 metabolises TAA to its hepatotoxic and highly reactive metabolite thioacetamide sulfoxide which covalently binds to the liver lipids and proteins, causing liver injury due to oxidative stress and systemic inflammation that mimic those seen in acute HE patients. HE is a severe neuropsychiatric syndrome with obvious symptoms such as anxiety, cognitive, memory, and learning impairment, balance problems, and personality changes. It may eventually lead to a coma and ultimately to death [2]. This deterioration of brain function is caused by the liver's inability to remove blood toxins, including ammonia and lipopolysaccharides, which causes systemic inflammation and activation of the circulatory neutrophils [3]. Then ammonia and other toxic agents go to the brain generating pathological changes such as neuroinflammation and neuropathy. Glutamine synthetase enzyme in brain astrocytes converts excess glutamate and ammonia into glutamine causing astrocytic swelling and cerebral edema due to its osmotic effect [4]. Fortunately, HE is a reversible disease and is caused by increased inflammation and oxidative stress in the brain. Therefore, using antioxidant scavengers and anti-inflammatory agents can be useful candidates for the treatment of HE. Gallic acid (GA) (3,4,5-trihydroxy benzoic acid) is a naturally occurring phenolic compound and is widely distributed in grapes, citrus fruits, sumac, green tea, different berries, oak bark, and gallnuts [5]. It is used as a food additive to prevent rancidity and oxidation of fats and oils in the industry [6]. Also, GA is used in numerous pharmaceutical and therapeutic applications due to its potent and diverse mechanisms of action such as anti-microbial, antiviral, antifungal, anticancer, anti-coagulant, and antioxidant activities [7]. In addition, it has antiepileptic, antianxiety, and antidepressant effects in animal models [2]. Via AMP-activated protein kinase activation, GA reduced inflammatory mediator expression and induced heme oxygenase-1 expression, which is an antioxidant enzyme involved in the suppression of oxidative stress and inflammation [8]. In a recent study, GA was effective in improving cognitive and memory disorders in addition to anxiety-like behaviors in rats with bile duct ligation-induced HE [2]. Because of its structure, it exhibits strong antioxidant properties, which indicates its ability to protect tissues and organs from oxidative stress. GA has been reported to prevent cognitive impairment induced by aluminum chloride and could be relevant in delaying the onset of Alzheimer’s disease. GA exerts its neuroprotective effects in several ways, including preventing deficits in neurotransmission, and oxidative stress in addition to lowering inflammatory cytokines (Tumor necrosis factor-α (TNF-α), interleukin-6 (IL-6), and interleukin-1β (IL-1β) and brain caspase-3 levels [9]. In the future, GA is suggested to be used as a promising pharmacological agent for preventing neuronal death and treating neurodegenerative diseases as it showed considerable neuroprotective effects in various in vitro and in vivo models [10]. Also, GA exhibited antioxidant and hepatoprotective effects against TAA-induced liver fibrosis in rats through regulation of the hepatic expression level of miR-21, miR-30, and miR-200 and inhibition of TGF-β1/Smad 3 signaling [5]. In addition, TAA-induced hepatic and renal toxicity in rats has been reported to be alleviated by GA because of its hypolipidemic effect in addition to the suppression of oxidative stress and inflammatory markers [11]. Based on a recent study, lipid metabolism improvement and liver enzyme amelioration are other helpful effects of GA against liver damage induced by methylglyoxal [12]. Further studies focusing on inflammatory cytokines and oxidative stress pathways are needed.

Metformin (Met) is a dimethyl biguanide that is widely used to treat diabetes mellitus (DM) but has also demonstrated pleiotropic effects. It has been reported that Met improved anxiety-like and depressive-like behaviors in addition to antioxidative activity in rats with cerebral ischemia-induced injury [13]. Additionally, Met has been known to reverse dyslipidemia and non-alcoholic fatty liver diseases by thought-provoking several antioxidant pathways and inhibiting inflammatory gene expression [14]. A previous study observed a lower prevalence of HE in rats taking Met due to a reduction in both glutaminase activity and ammonia production [15].

In this light, it has attracted attention that combining therapy could reduce the complications associated with acute HE via improvement of oxidative stress and inflammation in liver and brain toxicity. Various previous studies have revealed that a combination of GA and MET exhibited a more synergistic effect in diabetes management than each alone. A recent study provided that the combination of MET and GA demonstrated more effective renal protective effects than every single treatment via restoring AMPK/SIRT1 signaling and reducing oxidative stress [16]. Another study deduced that MET and GA co-administration in STZ-induced diabetic rats reduced inflammation, replenished glutathione, and modulate the JAK/STAT pathway which plays a critical role in the regulation of the liver. They deduced that combining GA and MET may have a stronger antioxidant effect, resulting in a more rapid decrease in pro-inflammatory cytokines [17]. Thus, these combinations have shown promising effects that could improve acute HE complications.

Inspired by these previous findings, we have taken a detailed mechanistic approach to explore the combined effect of GA and Met on the liver and brain damage associated with HE induced by TAA in rats. This is important because single therapy each alone may not be sufficient to help. Since every single therapy works by a different mechanism, we tried to find out if GA and Met co-administration potentiates their ameliorative effects on oxidative stress and inflammation pathways in TAA-induced acute HE in rats.

## Materials and methods

### Chemicals

TAA, GA, and Met were purchased from Sigma Chemical Co. (St. Louis, MO, USA). All other chemicals and reagents used were of analytical grade.

### Animals

Fifty healthy male albino rats weighing 200 ± 10 g were provided by the breeding unit of the National Organization for Drug Control and Research (NODCAR), Giza, Egypt. Under controlled conditions (21 ± 1°C constant temperature, humidity 55%, 12 h light–dark cycle), rats were housed and given a standard diet and water ad libitum. Animals were maintained for 2 weeks before the experiment as an acclimatization period.

### Experimental design

Five groups (10 animals each) of rats were used. Animals in the control group were given normal saline. Animals in the (TAA) group were only injected with a single intraperitoneal dose of 300 mg/kg TAA on the 25th day [4]. Animals in the (GA + TAA) group received a daily oral dose of GA (100 mg/kg/day) [7] and a single TAA dose on the 25th day. Animals in the (Met + TAA) group were given a daily oral dose of Met (200 mg/kg/day) [18] and a single TAA dose on the 25th day. (GA + Met + TAA) group: animals received a daily oral dose of both GA and Met (the same doses as before)) and single TAA dose on the 25th day. The GA and Met doses were chosen based on previous studies of no observable adverse effect level in rats [19, 20]. All the animals were euthanized on the 28th day by intraperitoneal injection of pentobarbital sodium (150 mg/kg body weight) and after 2 min, animals were sacrificed by decapitation [21]. Blood samples have been collected and the plasma, brain, and liver tissue were kept at − 20 °C for further analysis. Some of the brain and liver tissue samples were kept in formalin for histopathological examinations.

### Biochemical analysis

#### Determination of plasma biomarkers

The collected plasma was used to determine glucose, alanine aminotransferase (ALT), aspartate aminotransferase (AST), alkaline phosphatase (ALP), gamma-glutamyl transferase (GGT), alkaline phosphatase (ALP), total lipid profile, total bilirubin (TB), direct bilirubin (DB), albumin, total cholesterol (TC), triglycerides (TG), and ammonia (NH3), using commercial kits of Human, Biosystem, Spectrum, and Biomed diagnostics, Inc. Tumor necrosis factor-α (TNF-α), interleukin-6 (IL-6), and nuclear factor kappa β (NFkβ) were tested in plasma using rat ELISA kits supplied by Bioassay Technology Laboratory.

#### Antioxidant activity and oxidative stress

Liver tissues were homogenized in chilled 10 mM phosphate buffer solution (PBS) with optimal pH 7.4 and brain homogenate (1 g/10 ml, 75% aqueous methanol) was centrifuged and filtered. Both tissue homogenates were used to measure malondialdehyde (MDA) and glutathione (GSH) content using the high-performance liquid chromatography (HPLC) method [22, 23]. The sample was suspended in acetonitrile, homogenized and centrifuged 3000 g for 5 min. The clear supernatant was injected into HPLC using aminophase column for MDA while analytical column for GSH. HPLC is an accurate, sensitive and reproducible method that reflected the effect of induced oxidative stress on the levels of lipid peroxidation in cells. Due to the presence of the thiol group (-SH), GSH can interact with the Ellman′s reagent, with which it forms a reaction product through which the level of GSH can be quantified.

#### Determination of brain monoamine neurotransmitter levels

Norepinephrine (NE), dopamine (DA), and serotonin (5-HT) were determined by HPLC, using an Agilent column (ODS C18, 150 × 4.6 ID, 5 mm). The mobile phase consisted of 20 mM potassium phosphate solution (pH 2.7) and methanol in a 97%:33% ratio at a flow rate of 1.5 ml/min. Samples were injected at a volume of 25μL and a temperature of 25°c. The compounds were detected using a UV detector at 270 nm versus an external standard [24].

#### RNA extraction and RT-PCR analysis

Total hepatic RNA was extracted from 30 mg of frozen liver tissue using the QIAGEN RNeasy Mini Kit (Clinilab company, Egypt) according to the manufacturer’s instructions. The RNA concentration and purity were checked spectrophotometrically using the absorbance ratio at 260/280 nm. Ratios between 1.75 and 1.9 indicate pure RNA extract. The amplification cycling conditions were 10 min at 95 °C then 40 cycles of 15 s at 95 °C and 60 s at 60 °C. Reactions contained SYBR Green Master Mix (Applied Biosystems), gene-specific forward and reverse primers (10 μM), cDNA, and nuclease-free water. The data was then analyzed and quantified. The studied genes' relative expression was calculated using the comparative threshold cycle method and normalized to the ß- actin gene as the control housekeeping gene. The primers sequences used were designed using NCBI Primer Blast online tool and were as follows:

**Table:** 

	**Forward**	**Reverse**
**β-actin**	5ˋ-AGAGCTACGAGCTGCCTGAC-3ˋ	5ˋ-AGCACTGTGTTGGCGT ACAG-3ˋ
**FAS**	5ˋ-GCAGTGGCATGCTAAGTACC-3ˋ	5ˋ-AGTGGGGTTAGCCTGTGGAT-3ˋ
**CASP-3**	5ˋ -ATTATTCA GGCCTGCCGTGG-3ˋ	5ˋ-TGGATGAACCAGGAGCCATC-3ˋ

### Histopathological analysis

Hematoxylin and eosin (H&E) staining was performed on formalin-fixed, paraffin-embedded tissue samples (liver and brain) according to a method described previously [25].

### Statistical evaluation

The data are presented as mean ± SE. Comparisons between different groups were evaluated by one-way analysis (ANOVA) and Tukey’s multiple comparisons test using the software GraphPad InStat and Statistical Package for the Social Sciences program (SPSS version 20.0, Chicago, USA). A probability level of less than 0.05 was accepted as statistically significant [26].

## Results

### Effect on biochemical parameters

Our study observed that the administration of TAA showed an insignificant difference (*p* > 0.05) from the normal control group in fasting serum glucose level that was significantly (*p* = 0.0012) decreased only via the administration of GA + Met + TAA by 30% if compared to the TAA group as shown in Table 1. Also, our study indicated that TAA significantly (*P* < 0.001) increased liver dysfunction serum indices, including AST, ALT, GGT, ALP, DB, and TB levels by 1.5, 1.6, 2.8, 2.5, 1.7, and 3.3-folds compared to control animals. Treatment of rats by GA before TAA significantly (*P* < 0.05) decreased these TAA-induced changes in the previous parameters by 26, 31, 38, 37, 36, and 59%, respectively, versus the TAA group. While, treatment with Met before TAA significantly (*P* < 0.05) decreased them by 19, 19, 52, 27, 16, and 39%, respectively, versus the TAA group. However, the treatment with both GA and Met before TAA significantly (*P* < 0.05) decreased them by 28, 36, 47, 47, 40, and 64%, respectively, versus the TAA group. Similarly, our study found that TAA induction resulted in a significant decrease (*P* < 0.001) in the albumin level by 1.7-folds compared to the control group. Conversely, the treatment with GA or both GA and Met before TAA significantly (*P* < 0.05) increased them by 30 and 40%, respectively, versus the TAA group. Though administration of Met alone could not prevent TAA-induced decreases in the level of albumin significantly (*P* = 0.1334) when compared to the TAA group. Moreover, TAA significantly (*P* < 0.001) increased both TC and TG by twofold as compared to the normal control group. In contrast, pre-treatment with GA, Met, or both GA and Met significantly (*P* < 0.001) reduced TAA-induced elevated TC levels by 35, 12, and 40%, respectively, if compared to the untreated TAA group. On the contrary, the treatment with Met or both GA and Met before TAA significantly (*P* < 0.05) decreased the TG level by 22 and 35%, respectively, versus the TAA group. Though administration of GA could not prevent TAA-induced increases in the level of TG significantly (*P* = 0.7579) when compared to the TAA group. Results in Table 1 demonstrated that the TAA administration to rats induced a significant (*P* < 0.001) increase in ammonia level by 3.7-folds as compared with the control group. This rise was significantly (*P* < 0.001) attenuated by the administration of GA, Met, or both GA and Met by 24, 21, and 26%, respectively, compared to the TAA control group.

**Table 1:** Effect of a single dose of TAA alone or after GA and/or Met on levels of some related biochemical parameters

Groups	GA + TAA	MET + TAA	GA + Met + TAA	TAA	Control
Glucose (mg/dL)	150.4 ± 8.02	130.8** ± **7.92	110.5** ± **9.14^b^	159.8** ± **11.8	133.9** ± **3.50
AST(U/l)	148.9 ± 5.52^b^	162.4 ± 2.28^ab^	145.1 ± 1.90^b^	200.9 ± 5.00^a^	131.3 ± 7.69
ALT(U/l)	71.38 ± 2.31^b^	83.57** ± **5.42^ab^	65.98** ± **2.57^b^	103.6** ± **5.77^a^	63.28** ± **0.83
GGT(u/l)	11.16** ± **0.11^ab^	8.675** ± **2.94^ab^	9.669** ± **0.10^ab^	18.08** ± **0.12^a^	6.427** ± **0.25
ALP(U/l)	559.1** ± **11.9^ab^	645.9** ± **22.0^ab^	475.3** ± **15.15^ab^	890.1** ± **26.85^a^	357.9** ± **8.30
DB (mg/dL)	0.168 ± 0.01^b^	0.221 ± 0.01^ab^	0.158 ± 0.00^b^	0.264 ± 0.02^a^	0.154 ± 0.00
TB (mg/dL)	0.540** ± **0.04^b^	0.800** ± **0.08^ab^	0.477** ± **0.03^b^	1.317** ± **0.13^a^	0.400** ± **0.01
Albumin (g/dL)	3.302 ± 0.02^ab^	3.029 ± 0.01^a^	3.553 ± 0.05^ab^	2.535 ± 0.21^a^	4.247 ± 0.27
TC (mg/dL)	100.4** ± **4.89^ab^	137.5** ± **6.42^ab^	93.03** ± **6.50^ab^	155.6** ± **1.05^a^	74.84** ± **1.33
TG (mg/dL)	111.6 ± 6.80^a^	93.76 ± 8.22^ab^	78.84 ± 3.29^b^	120.6 ± 5.08^a^	61.38 ± 4.64
NH3 (μM/L)	234.9 ± 3.16 ^ab^	245.05 ± 2.16^ab^	224.78 ± 2.8^ab^	305.88 ± 3.24^a^	81.98 ± 1.9

The data are presented as mean ± SE; *n* = 6

*GA* gallic acid, *Met* metformin, *TAA* thioacetamide, *ALT* alanine transaminase, *AST* aspartate transaminase, *ALP* alkaline phosphatase, *GGT* gamma-glutamyl transferase, *DB* direct bilirubin, *TB* total bilirubin, *TC* total cholesterol, *TG* triglycerides, and *NH3* blood ammonia level

^a^ is significantly different from control group at *p* < 0.05 and ^b^ is significantly different from TAA group, at *p* < 0.05

### Effect on inflammatory biomarkers

**Fig. 1:**
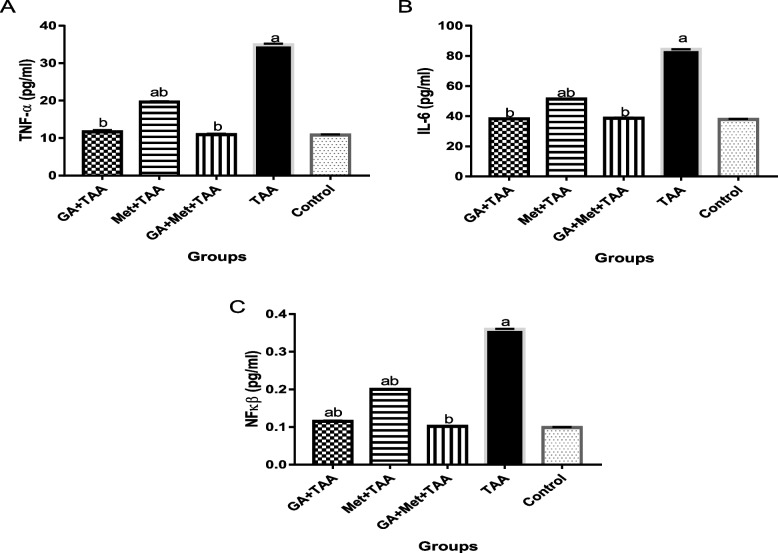
Effect of a single dose of TAA alone or after GA and/or Met on some inflammatory markers; (**A**) TNF-α, (**B**) IL-6, and (**C**) NFkβ. The data are presented as mean ± SE; *n* = 6 for each group. ^a^ is significantly different from the control group at *p* < 0.05 and ^b^ is significantly different from the TAA group, at *p* < 0.05. (GA) gallic acid, (Met) metformin, (TAA) thioacetamide, (TNF-α) Tumor necrosis factor-α, (IL-6) interleukin-6, and (NFkβ) nuclear factor kappa β

Data in Fig. 1 also revealed a significant (*p* < 0.0001) increase in levels of the inflammatory markers TNF-α, IL-6, and NFkβ in the TAA group (3.2, 2.2, and 3.6-fold, respectively) as compared to the control group. Administration of GA significantly (*P* < 0. 0.0001) decreased these inflammatory markers by 67, 54, and 68%, respectively, versus the TAA group. While, treatment with Met significantly (*P *< 0. 0.0001) decreased them by 44, 39, and 44%, respectively, versus the TAA group. Rats administered both GA and Met showed a pronounced reduction (*P* < 0.0001) decreasing them by 69, 54, and 72%, respectively, versus the TAA group.

### Effect on oxidative stress

**Fig. 2:**
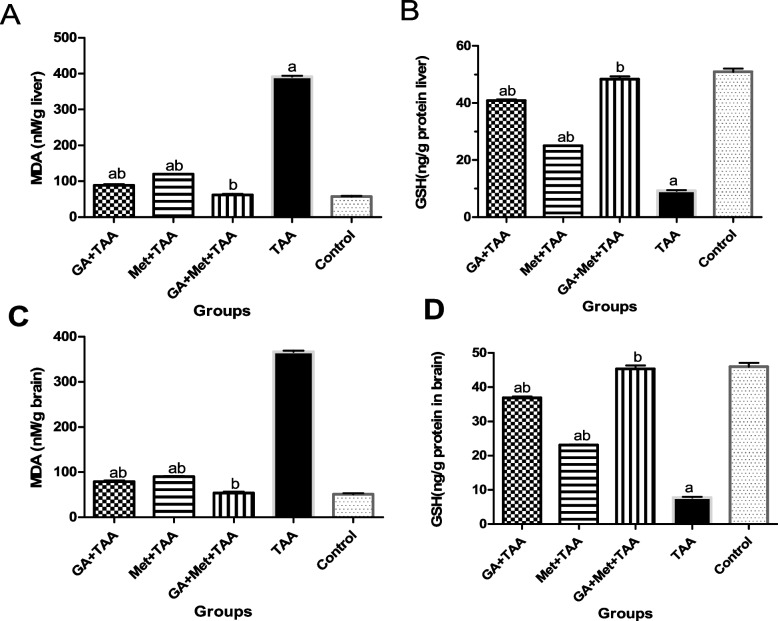
Effect of a single dose of TAA alone or after GA and/or Met on oxidative stress; (**A**) MDA in the liver, (**B**) GSH in the liver, (**C**), MDA in the brain, and (**D**) GSH in the brain. The data are presented as mean ± SE; *n* = 6 for each group. ^a^ is significantly different from the control group at *p* < 0.05 and ^b^ is significantly different from the TAA group, at *p* < 0.05. (GA) gallic acid, (Met) metformin, (TAA) thioacetamide, (MDA) malondialdehyde, and (GSH) glutathione

Regarding the oxidative markers, TAA significantly (*P* < 0.0001) increased the hepatic and brain MDA content by 5.5 and 7.2-fold, respectively, but caused a marked decrease (*P* < 0.0001) in their GSH content by 5.5 and sixfold, respectively, versus control animals. Though GA treatment significantly (*P* < 0.0001) decreased the hepatic and brain MDA content by 4.4 and 4.7-fold, respectively, while increased their GSH content by 4.4 and 4.8-fold, respectively versus the untreated TAA group. Also, Met caused a significant decrease (*P* < 0.0001) in the hepatic and brain MDA content by 3.3 and fourfold, respectively, while increasing their GSH content by 2.7 and threefold, respectively, compared with the untreated TAA group. However, pre-treatment of both drugs decreased hepatic and brain MDA content significantly (*P* < 0.0001) by 6.3 and 6.8-fold, respectively, and increased their GSH content by 5.2 and 5.9-fold, respectively, compared with the untreated TAA group (Fig. 2 A-D).

### Effect on some brain neurotransmitters

**Fig. 3:**
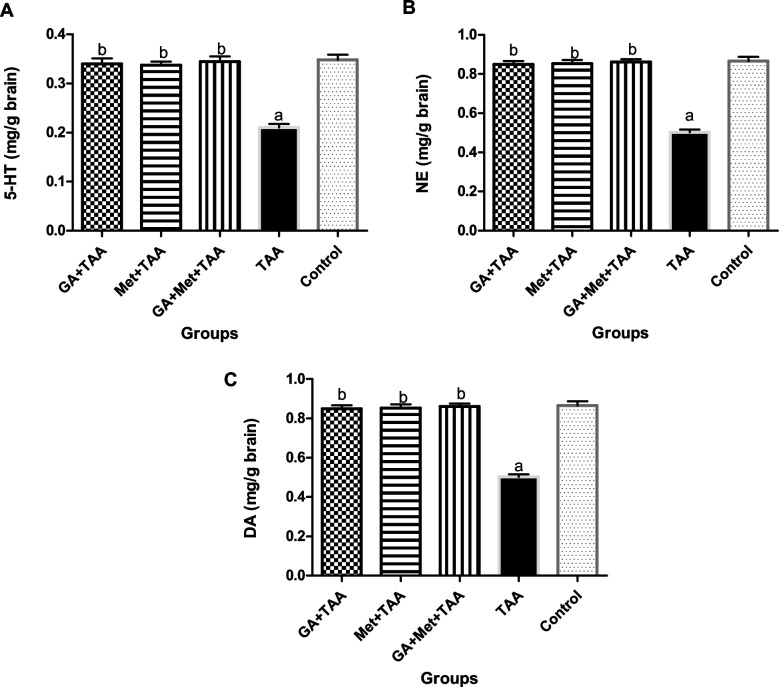
Effect of a single dose of TAA alone or after GA and/or Met on levels of some brain neurotransmitters; (**A**) 5-HT, (**B**) NE, and (**C**) DA. The data are presented as mean ± SE; *n* = 6 for each group. ^a^ is significantly different from the control group at *p* < 0.05 and ^b^ is significantly different from the TAA group, at *p* < 0.05. (GA) gallic acid, (Met) metformin, (TAA) thioacetamide, (5-HT) serotonin, (NE) norepinephrine, and (DA) dopamine

Moreover, TAA significantly (*P* < 0.05) decreased some brain neurotransmitters, including 5-HT, NE, and DA by 40, 42, and 52%, respectively, as compared to the normal control group. In contrast, pre-treatment with GA significantly (*P* < 0.001) increased their levels by 62%, 69, and 87%, respectively, if compared to the untreated TAA group, as shown in Fig. 3. Moreover, pre-treatment with Met significantly (*P* < 0.001) increased them by 60, 70, and 81%, respectively, compared to the untreated TAA group. Furthermore, the combination of both GA and Met increased them by 64, 72, and 100%, respectively, when compared to the untreated TAA group.

### Effect on some apoptotic genes

**Fig. 4:**
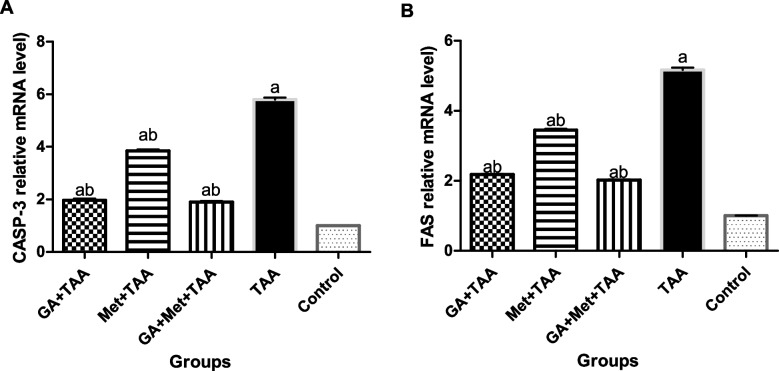
Effect of a single dose of TAA alone or after GA and/or Met on the expression of some apoptotic genes; (**A**) CASP-3, and (**B**) FAS. The data are presented as mean ± SE; *n* = 6 for each group. ^a^ is significantly different from the control group at *p* < 0.05 and ^b^ is significantly different from the TAA group, at *p* < 0.05. (GA) gallic acid, (Met) metformin, (TAA) thioacetamide, (CASP-3) caspase-3, and (FAS) death receptor

Our study revealed that a single dose of TAA showed a significant (*P *< 0.001) up-regulation of the gene expression of CASP-3 and FAS in the liver by 5.8 and 5.1-folds, respectively, compared to the control group. In contrast, the administration of GA produced a significant down-regulation (*P* < 0.01) of these genes by 66 and 58%, respectively. The effect of Met is less but significant (*P* < 0.01); 34 and 33, respectively. As regards the combined treatment (GA and Met), it up-regulated them by 67% and 61%, respectively in a significant way (*P* < 0.01), if compared to the untreated TAA group (Fig. 4).

### Effect on histopathological examination

**Fig. 5:**
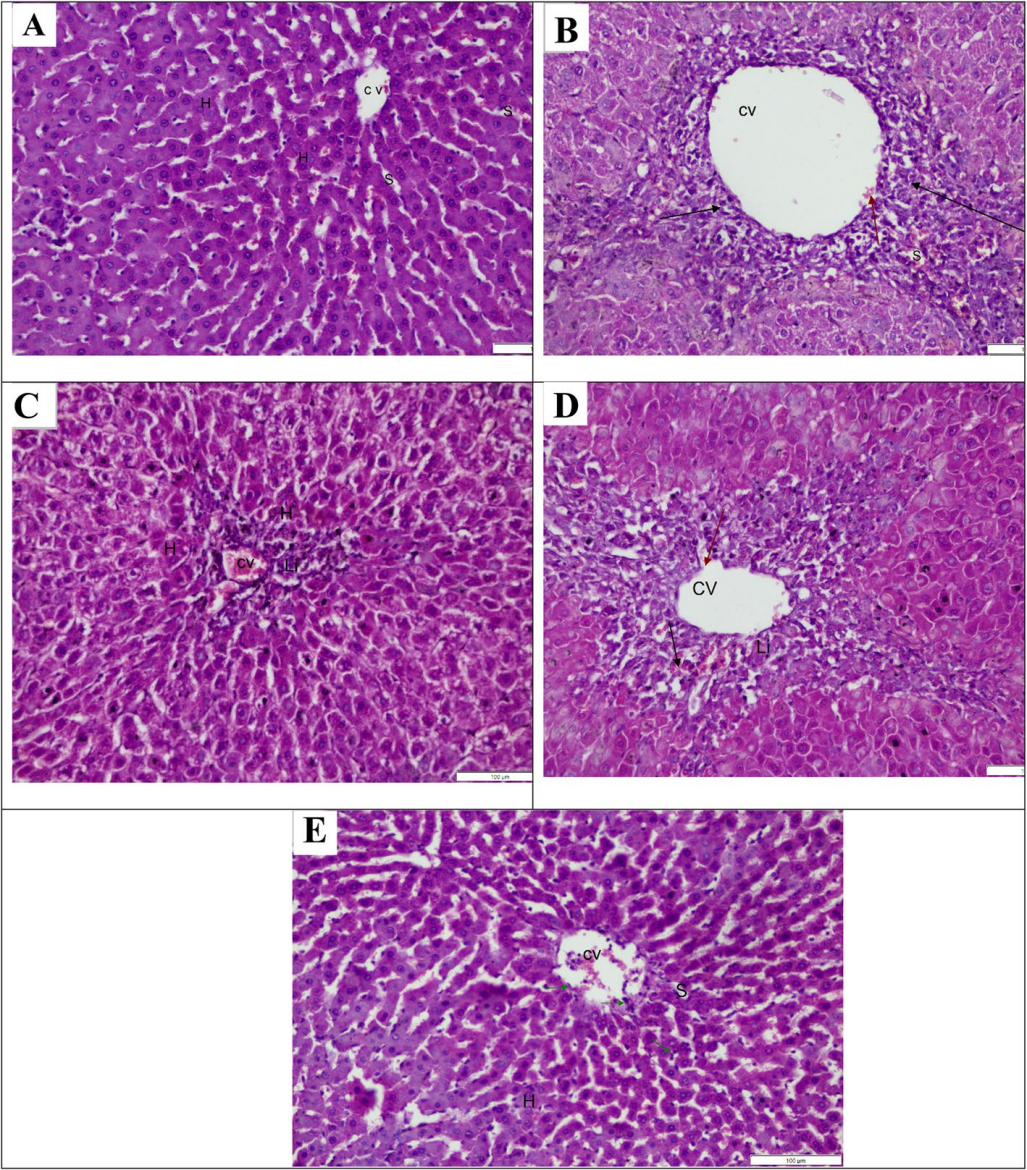
Effect of TAA and/or (GA and/or Met) on histopathological examination of livers (H&E 100 μm). **A** photomicrograph of a section in the liver of a control animal, displaying normal central vein (CV) and normal hepatocytes (H) with intact sinusoids (S). **B**-A photomicrograph of a section in the liver of the TAA group, displaying widening of the central vein (CV), loss of cellular structure around the central vein (black arrow) and sinusoids oozing blood (S). **C**-A photomicrograph of a section in the liver of animals treated with TAA and GA, displaying a central vein filled with blood (CV), leukocyte infiltration around the central vein (LI) and hepatocytes with pyknotic nucleus (H). **D**-A photomicrograph of a section in the liver of animals treated with TAA and Met, displaying widening of central vein (CV) with detachment of endothelial lining of c.v (red arrow), leukocyte infiltration around the central vein (LI), and sinusoids oozing blood (black arrow). **E**-A photomicrograph of a section in the liver of animals treated with TAA (GA + Met), displaying nearly normal hepatic architecture with intact hepatocytes (H) radiated from an apparently normal central vein (CV), and apparently normal sinusoids(S) with numerous Kupffer cells (green arrow). (GA) gallic acid, (Met) metformin, (TAA) thioacetamide

A single injection of TAA significantly affected liver morphology and structure (Tables 2 and 3). Liver sections from rats of the TAA group showed a severe degree of centrilobular hepatic necrosis, fibrosis, congestion, vacuolation, and hyperplasia (Fig. 5B) when compared to the normal group which showed normal hepatocytes (Fig. 5A). The liver of animals treated with TAA and GA show central vein congestion associated with a fatty change in a few individual hepatocytes and inflammatory cell infiltration in the portal area (Fig. 5C). The sections of liver from rats of the (TAA and Met) group showed a fatty change in a few individual hepatocytes with congestion in the central vein and a marked decrease of hepatic and especially periportal necrosis (Fig. 5D). The liver of animals treated with TAA and both (GA + Met) showed a marked decrease in portal vein congestion and periductal fibrosis with intact hepatocytes parenchyma (Fig. 5E).

**Table 2:** Histological scores in the liver of different groups

	Lobular inflammation and necrosis	Portal inflammation and necrosis	fibrosis
Control	0	0	0
TAA	3	3	3
Met + TAA	2	1	1
GA + TAA	1	1	1
Met + GA + TAA	0	1	0

See Table 3 for scoring descriptions

*GA* gallic acid, *Met* metformin, *TAA* thioacetamide

**Table 3:** Ludwig system for grading hepatic inflammation and fibrosis [27]

Lobular inflammation and necrosis	Portal inflammation and necrosis	fibrosis	Activity description	Score
None	None	None	No activity	0
spotty	Patchy	Portal fibrosis	Minimal	1
little hepatocellular damage	little portal tract damage	Periportal fibrosis	mild	2
noticeable hepatocellular change	noticeable change in all portal tract	Septal fibrosis	Moderate	3
diffuse hepatocellular damage	Bridging necrosis	Cirrhosis	Sever	4

**Fig. 6:**
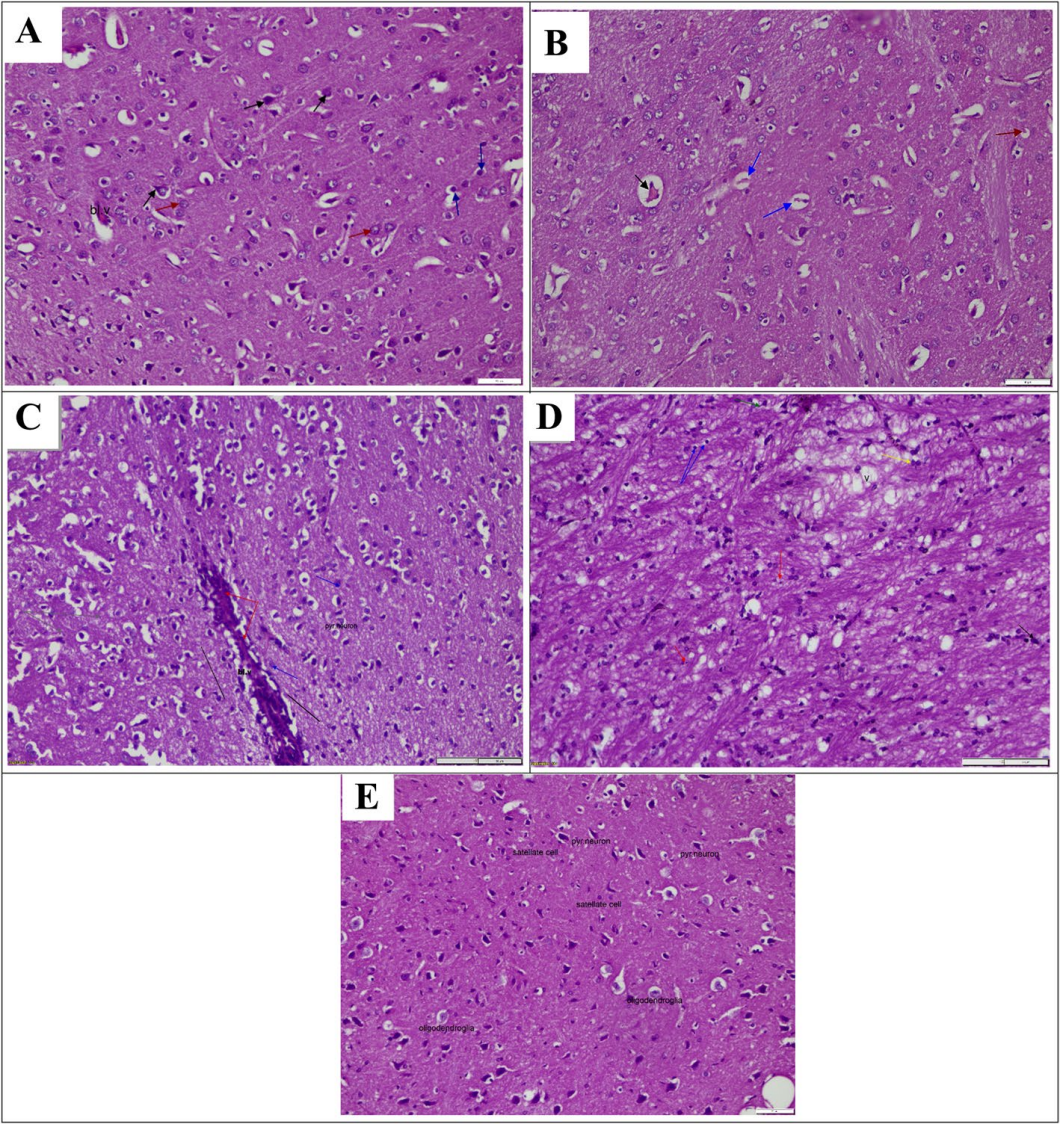
Effect of TAA and/or (GA and/or Met) on histopathological examination of brains (H&E.100 μm). **A**-A photomicrograph of a section in the cerebrum of a control animal, displaying normal architecture with intact blood vessels (bl.v), pyramidal neurons (black arrow), satellite cells (red arrow), and oligodendroglia (blue arrow). **B**-A photomicrograph of a section in the cerebrum of affected TAA group, displaying bulged neuron (black arrow), neurons shrinkage (blue arrow) and a pyknotic nucleus (red arrow). **C**-A photomicrograph of a section in the cerebrum of animals treated with TAA and GA, displaying large pyramidal cells without dendrites (black arrow), axons of small pyramidal cells (green arrow), vacuoles in the cerebral cortex with disarrangement of cerebral cells (v), granular cells (red arrow), small pyramidal cells without dendrites (yellow arrow), and numerous micoglial cells (blue arrow). **D**-A photomicrograph of a section in the cerebrum of animals treated with TAA and Met, displaying pyramidal neuron appear normal, relaxation of waves between neuron (black line), small pyramidal neurons (green arrow), large pyramidal neurons (blue arrow), foamy structure between neurons (grey arrow) and numerous microglial cells (red arrow). E-A photomicrograph of a section in the cerebrum of animals treated with TAA (GA + Met) showed an normal histological structure of the brain. (GA) gallic acid, (Met) metformin, (TAA) thioacetamide

Figure 6 and Tables 4 and 5 show the cerebrum section of the brain of experimental animals after administration of TAA, GA, and Met. A significant bulged neuron, neurons shrinkage, and a pyknotic nucleus were observed in the TAA group (Fig. 6B) when compared with the negative control which showed normal architecture with intact blood vessels, pyramidal neurons, satellite cells, and oligodendroglia (Fig. 6A). However, co-administration of TAA with GA displays large pyramidal cells without dendrites, axons of small pyramidal cells, vacuoles in the cerebral cortex with disarrangement of cerebral cells, small pyramidal cells without dendrites, and more microglial cells (Fig. 6C). Also, treatment with TAA and Met, displays pyramidal neurons appear normal, relaxation of waves between neurons, small pyramidal neurons, large pyramidal neurons, the foamy structure between neurons, and numerous microglial cells (Fig. 6D). While treatment with both GA and Met in addition to TAA shows a nearly normal histological structure of the brain (Fig. 6E).

**Table 4:** Histological scores in the liver of different groups

	Meningeal cellular proliferation	Neuronal degeneration	Focal gliosis	Congested blood vessels
Control	0	0	0	0
TAA	3	3	3	3
Met + TAA	2	3	2	2
GA + TAA	1	2	2	2
Met + GA + TAA	0	1	1	0

See Table 5 for scoring descriptions

*GA* gallic acid, *Met* metformin, *TAA* thioacetamide

**Table 5:** Scoring of the histopathological alterations in the brain [28]

Activity description	Score
Nil histopathological alteration	0
Minimal	1
mild	2
Sever	3

## Discussion

Combination therapy in HE becomes essential because of the previously mentioned complications of HE that arise from several pathways defects. There is some evidence that herbs may be administered along with conventional drugs, and this may enhance the possibility of herb-drug antioxidant activity. Surprisingly, the combination therapy of Met and GA has been used recently for treating DM several times before due to the improvement of antioxidant status and amelioration of inflammation [17, 29, 30]. Another study demonstrated that DM may increase systemic inflammation and serum ammonia, which enhances the development of HE as a result [31]. Moreover, Met regulates ammonia homeostasis due to modulating glutamine metabolism in the model of HE in a previous study [15]. Long-term MET therapy improved neurobehavioral disorders (cognitive, anxiety, and depressive-like behaviors) and could be used for the treatment of brain ischemic conditions [13]. In addition, GA reduced anxiety-like behaviors and improved memory, learning, and motor activity in bile duct ligation-induced HE [2]. Based on all previous data, we hypothesized that adding GA to patients taking Met would decrease the incidence of HE.

In our study, the results showed that TAA induced a state of hepatic damage accompanied by brain affection. TAA induced hepatotoxicity through elevation of liver function enzymes (ALT, AST, GGT, and ALP), bilirubin, and ammonia levels in addition to a profound reduction in albumin level compared to the normal group. These cytosolic enzymes are the best indicator of liver necrosis, as their elevations reflect a leakage in the cell membrane, which is linked to the death of hepatocytes [32]. These results were in agreement with previous results [33–35]. They proposed that the resulting hyperammonemia may cause more liver insufficient detoxification and further decreased ammonia urinary loss due to alkalosis, leading to direct ammonia-induced neurotoxicity. A previous study suggested a new mechanism of brain dysfunction in patients with liver diseases that resulted in neuronal dysfunction and cognitive impairment [36]. The glymphatic system is a newly discovered brain-wide pathway for waste clearance in the central nervous system of vertebrates. Thus a defect in this glymphatic system leads to an abnormal accumulation of soluble proteins, waste products, and excess extracellular fluid formed in the brain during its action, which can trigger neuroinflammation and neurocognitive disorders. Additionally, TAA-induced intoxication caused innate antioxidant system impairment, as evidenced by a marked consumption of both brain and liver GSH content while an increase in their MDA content. This observation is in agreement with many earlier studies which proved the TAA-induced lipid peroxidation and oxidative stress effect [18, 32, 37]. In harmony with the current results, a previous study suggested that reactive oxygen species (ROS) produced by TAA induce inflammation via the activation of Nuclear factor kappa B (NF-κB), which subsequently increased the release of downstream proinflammatory mediators such as IL-6 and TNF-α [34]. Thus, oxidative damage can cause neuroinflammation by increasing these proinflammatory cytokines resulting in certain changes in brain neurotransmission associated with cognitive impairment. Moreover, the findings of the present study showed that a significant decrease in some neurotransmitter levels such as 5-HT, NE, and DA was observed in the brains of TAA animals. Together, these findings intensely propose co-interaction between ammonia, ROS, and inflammatory cytokines in the brain of the TAA group in this study, proving a previously close relationship between oxidative stress and inflammation, and cognition dysfunction [35]. Our results evidenced that TAA induced an up-regulation of both CASP-3 and FAS expressions in liver tissues, suggesting TAA proapoptotic activity. Together, these interpretations about TAA different activities powerfully recommend a synergistic interaction and crosstalk between oxidative stress, inflammation and apoptosis. Such observation is in agreement with robust evidence described previously for the presence of two pathways; intrinsic apoptotic pathway via oxidative imbalance generation and extrinsic apoptotic pathway via hepatic Kupffer cells activation to produce several regulatory factors for liver regeneration and cellular immune response. TNF-α and IL-6 cytokines are the most significant regulatory molecules for phase I of liver regeneration [33]. On the same line, histopathological results confirmed the acute injury in both liver and brain tissue. Severe hepatocellular necrosis, inflammation and fibrosis were observed after TAA administration. Brain sections in TAA group exhibited neuroinflammation, edema and necrotic cell structures. Similar findings have been previously revealed by various studies [31, 36, 37].

Oxidative stress plays a major role in inflammatory processes via the activation of a variety of transcription factors leading to the differential expression of some genes involved in inflammatory pathways. NF-κB, a major redox-sensitive transcription factor and a master regulator of the inflammatory response, consists of two subunits. Its most common form is the p65/p50 heterodimer and binds to the IκB proteins in the cytoplasm as an inactive form. ROS cause the degradation of IκB proteins in the cytoplasm when they phosphorylate them through the inhibitor of the NF-B kinase (IKK) enzyme. Thus, NF-κB is released, activated, and translocates to the nucleus to induce the expression of several molecules, such as TNF-α, IL-1β, and IL-6, which are involved in the inflammatory process [38]. Also, NF-kB plays an important role as a signaling pathway in the central nervous system, activating survival cascades in response to neuronal damage. The major problem occurs when the tissue needs a high cellular turnover because the inflammatory cascade targets cellular proliferation and induces apoptosis. The transcription factor NF-kB regulates the expression of anti-apoptotic proteins [39]. Apoptosis initiation through p53 activation occurs in several ways, one of which induces FAS mRNA and translocation of the synthesized FAS to the cell surface. In addition, p53 acts directly on the outer mitochondrial membrane, and once inside the organelle, it interacts with B-cell lymphoma-2 (Bcl-2), leading to the release of cytochrome C and activation of CASP-3.

On the contrary, the results of the current study indicated that GA and/or Met significantly reversed the oxidative stress level, and the apoptotic and inflammatory markers induced by TAA administration in both the brain and liver. Interestingly, many studies recommended that the hepatoprotective effect protects against neurotoxicity through antioxidant and anti-inflammatory actions that are exhibited here by GA and Met [33, 40]. The hepatoprotective effect of GA and/or Met against TAA-induced hepatotoxicity was revealed significantly via reducing serum levels of ALT, AST, GGT, and ALP, compared to the TAA group. The prevention of the liver enzyme elevation indicates the preservation of the integrity of the hepatic tissues that may be attributed, at least in part, likely due to the widely known antioxidant activities of GA and Met. For instance, the antioxidant activity of GA was previously demonstrated in the diminution of these cytosolic enzymes in experimentally TAA-induced liver fibrosis [5].

It was also reported in a recent study that GA was able to decline the exudation of these enzymes and scavenge ROS that are produced during the cell membrane injury induced by paraquat in male rats [41]. The antioxidant effect could be linked to the phenolic hydroxyl groups in the GA structure, especially at the para position to the carboxylic group, which have a potent scavenging effect for the free radicals as previously mentioned before [42]. Moreover, GA caused a significant decrease in TC level while a nonsignificant decrease in TG was in parallel with a preceding study [43]. At this point, they illustrated that hypolipidemic effects of GA may be due to the interruption of enzymes and protein’s function involved in lipoprotein metabolism pathways, fat absorption reduction, and enhancement of both cholesterol excretion into feces and cholesterol secretion through bile. Following our results, GA treatment previously counterbalanced TAA-induced liver damage as indicated by reduced lipid peroxidation and aminotransferases levels together with normalized GSH content. A recent study has shown the inhibitory effect of GA to prevent NF-κB activation and reduce inflammatory cytokines productions and thus inflammation, in harmony with our outcomes [44]. They added that NF-κB also boosts NADPH oxidase expression as a source of endogenous free radicals, leading to oxidative stress. In parallel with a prior study, GA played an anti-apoptotic role by regulating some pro-apoptotic markers such as CASP-3 protein expression [45]. Based on our findings, we can imply that GA can significantly modulate the monoaminergic system by increasing the neurotransmitters levels and thus exerting a neuroprotective effect [46].

Likewise, Met, an inexpensive and broadly available medicine, has been shown to protect hepatocytes from oxidative stress-induced apoptosis in previous research [18]. Our study findings agree with previous data and also found that Met significantly decreased AST, ALT, ALP, GGT, total bilirubin, and direct bilirubin levels [47]. They mentioned that Met possibly exerts its protective effects by several mechanisms of action as decreasing the mitogen-activated protein kinase and CASP-3 activities, alleviating oxidative stress, NF-κB activation, apoptosis inhibition, and mitochondrial dysfunction. In line with our results, treatment with Met in a previous study improved TC and TG in addition to the normalization of glucose [48]. They reported that this could be attributable to decreasing blood glucose levels and hepatic gluconeogenesis while increasing glucose uptake into muscles and thus increasing fatty acid oxidation in adipose tissue. This was associated with improvement of dyslipidemia and reverse fatty liver, probably through reduced pro-inflammatory cytokines production, and hence hepatic inflammation. Met has been previously demonstrated to have the ability to regulate NF-κB, resulting in the reduction of TNF-α and IL-1β levels and thus showing anti-inflammatory effect [49]. Met, in a former study, reduced palmitate-induced apoptotic cell death by reducing the caspase-3/7 activity at low doses [50].

Also, in an earlier study, this anti-diabetic drug decreased CASP-3 expression in the liver tissue of streptozotocin-induced diabetic rats in harmony with our findings. Furthermore, they emphasized that Met protected the hippocampus and improved learning and memory impairment due to its antihyperglycemic, anti-oxidative, anti-inflammatory, and anti-apoptotic mechanisms [51]. These outcomes are in harmony with our and others' findings that shed light on the neuroprotective effect of Met to improve cognitive impairment in diabetic epileptic rats by restoring the altered brain neurotransmitters glutamate and γ-aminobutyric acid [52]. The histopathology results supported and confirmed these aforementioned results as the liver tissues of GA and Met groups displayed histology scores normalization, nearly-looking hepatic and brain architecture, in harmony with earlier studies [42, 53]. Also, recent studies showed fewer necrotic cells and normal-looking neurons and glial cells after treatment with GA or Met, in agreement with our results [54, 55].

To the best of our knowledge, this is the first study to report the hepatoprotective and cognitive enhancing effect of GA and Met combination against acute liver and brain injury induced by TAA in rats, focusing on their modulatory effects on NF-κB signaling pathway. Combination therapies are becoming increasingly popular, as they improve clinical outcomes by targeting a multitude of pathways with the potential likelihood of having synergistic effects [34]. From the first look, GA and Met efficiently ameliorated TAA-induced acute liver and brain injury and reduced the liver and brain MDA content with GSH stores replenishment in addition to decreasing pro-inflammatory cytokines production and CASP-3 and FAS gene expressions. Treatment with GA and Met combination resulted in a prominent improvement of HE complications, relative to monotherapy suggesting that both agents potentiated the antioxidant anti-inflammatory and anti-apoptotic effects of each other. That this mixture may be one of the most powerful approaches for the treatment of acute liver and brain toxicity. Since MET is a widely used drug with few adverse effects, it has the potential to be administered in the clinic for the treatment of HE. Combination therapy has become one of the effective therapies to manage patients with HE, especially with patients who are at a higher risk of experiencing HE-related complications such as cognitive and anxiety-like behaviors.

## Conclusion

According to the information provided by our study, these combinatorial therapies could be useful in HE management and reliving its complication. Additional studies, including clinical trials, are encouraged to conclude the overall potentiating effect of GA on Met in HE management and produced in the pharmaceutical industries.

## Data Availability

Additional data will be accessible from the corresponding author, drehsankhedre@hotmail.com.

## Abbreviations

GA: Gallic acid
Met: Metformin
HE: Hepatic encephalopathy
TAA: Thioacetamide
CASP-3: Caspase-3
DM: Diabetes mellitus
MDA: Malondialdehyde
GSH: Glutathione
HPLC: High-performance liquid chromatography
NE: Norepinephrine
DA: Dopamine
5-HT: Serotonin
H&E: Hematoxylin and eosin
SPSS: Statistical Package for the Social Sciences program
TNF-α: Tumor necrosis factor-α
IL-6: Interleukin-6
IL-β: Interleukin-1β
NFkβ: Nuclear factor kappa β
ALT: Alanine transaminase
AST: Aspartate transaminase
ALP: Alkaline phosphatase
GGT: Gamma-glutamyl transferase
DB: Direct bilirubin
TB: Total bilirubin
TC: Total cholesterol
TG: Triglycerides
NH3: Blood ammonia level
